# Sustainable Plant-Based Biopolymer Membranes for PEM Fuel Cells

**DOI:** 10.3390/ijms232315245

**Published:** 2022-12-03

**Authors:** Songtao Li, George Cai, Songze Wu, Aniket Raut, William Borges, Priyanka R. Sharma, Sunil K. Sharma, Benjamin S. Hsiao, Miriam Rafailovich

**Affiliations:** 1 Department of Material Science and Chemical Engineering, Stony Brook University, Stony Brook, NY 11794, USA; 2 Department of Chemistry, Stony Brook University, Stony Brook, NY 11794, USA

**Keywords:** carboxycellulose, citric acid, PEMFCs, proton conductivity, tensile strength, nanopapers

## Abstract

Carboxycellulose nanofibers (CNFs) promise to be a sustainable and inexpensive alternative material for polymer electrolyte membranes compared to the expensive commercial Nafion membrane. However, its practical applications have been limited by its relatively low performance and reduced mechanical properties under typical operating conditions. In this study, carboxycellulose nanofibers were derived from wood pulp by TEMPO oxidation of the hydroxyl group present on the C6 position of the cellulose chain. Then, citric acid cross-linked CNF membranes were prepared by a solvent casting method to enhance performance. Results from FT-IR spectroscopy, ^13^C NMR spectroscopy, and XRD reveal a chemical cross-link between the citric acid and CNF, and the optimal fuel cell performance was obtained by cross-linking 70 mL of 0.20 wt % CNF suspension with 300 µL of 1.0 M citric acid solution. The membrane electrode assemblies (MEAs), operated in an oxygen atmosphere, exhibited the maximum power density of 27.7 mW cm^−2^ and the maximum current density of 111.8 mA cm^−2^ at 80 °C and 100% relative humidity (RH) for the citric acid cross-linked CNF membrane with 0.1 mg cm^−2^ Pt loading on the anode and cathode, which is approximately 30 times and 22 times better, respectively, than the uncross-linked CNF film. A minimum activation energy of 0.27 eV is achieved with the best-performing citric acid cross-linked CNF membrane, and a proton conductivity of 9.4 mS cm^−1^ is obtained at 80 °C. The surface morphology of carboxycellulose nanofibers and corresponding membranes were characterized by FIB/SEM, SEM/EDX, TEM, and AFM techniques. The effect of citric acid on the mechanical properties of the membrane was assessed by tensile strength DMA.

## 1. Introduction

With mounting global energy demands projected to increase by 50% or more by the next decade, humans are using natural petroleum at a rate reported to be 105 times faster than nature can provide [1,2]. Such dependence on fossil fuels is not only unsustainable but also leads to climate change from increasing greenhouse gas levels in the atmosphere [3]. Since it was proposed that global warming can be slowed and perhaps reversed only when society replaces fossil fuels with renewable, carbon-neutral alternatives, the search for “clean” energy has become imperative [4]. Today, various renewable energy systems, such as photovoltaics [5], wind [6], geothermal [7], and biomass [8], have been intensively studied and extensively applied around the world. Among them, fuel cells, which convert chemical energy directly into electrical energy, are proposed as a promising alternative energy medium due to their high efficiency and low to nonexistent emissions [9,10].

Among various types of fuel cells, proton exchange membrane fuel cells (PEMFCs) are versatile energy conversion devices because of their less extreme operating conditions, efficient power conversion, and utility in transportation vehicles [11,12,13]. Thus, PEMFCs have been developing most rapidly in the past decades [14]. Nafion, a perfluorinated sulfonic acid polymer produced by Du Pont, is a good proton conductor for hydrated membranes with long-term electrochemical stability and high mechanical strength [13,15]. However, Nafion membranes suffer from decreased conductivity and stability at high temperatures, excessive hydrogen crossover, and a prohibitively high cost of up to USD 800/m^2^ [10,12,16,17]. Therefore, several alternative materials with high performance and relatively lower cost have been developed as potential proton exchange membranes (PEMs).

Cellulose, the main component in cell walls of plants, algae, and bacteria, is the most abundant biopolymer on the planet and has seen application in various problems relating to sustainability, including energy and water purification, because it is renewable, biocompatible, cheap, naturally biodegradable, and chemically stable [18,19,20,21,22,23,24,25,26,27,28,29,30,31,32]. Recently, nanoscale cellulose materials have gained much interest thanks to their dimensional stability, low thermal expansion coefficient, nanoscale morphology, chemically tunable surface functionalities, ability to be obtained in various dimensions, and renewability [33,34]. According to the nomenclature proposed by the Technical Association of the Pulp and Paper Industry (TAPPI), nanocellulose can be classified into two main subcategories, cellulose nanocrystals (CNCs) and cellulose nanofibers (CNFs), based on size and aspect ratio [35]. Nanocellulose has been applied in PEMs due to its low cost, excellent gas barrier properties, and acidic carboxyl functional groups [36]. A pilot plant established by the University of Maine and the US Department of Agriculture Forest Service has been able to produce CNC on a commercial scale with sales price estimated by TAPPI to be as low as $4/lb [37], including the costs of raw materials and production process, which is likely to further decrease with an increased scale of production. Previously, both cellulose and nanocellulose have been used extensively as an additive to enhance the performance of Nafion and other conductive polymers [12,38,39,40,41,42].

While the innate proton conductivity of nanocellulose is relatively low [36], various methods have been employed to enhance proton conductivity in nanocellulose-based materials. For example, Smolarkiewicz et al. prepared a nanocellulose film doped with imidazole as a “dry” electrolyte that exhibits nearly four orders of magnitude higher conductivity than a pure cellulose sample while maintaining thermal stability from 110 °C to 150 °C [43]. Bideau et al. synthesized a conductive nanocellulose-based film through grafting N-(3-aminopropyl)pyrrole onto oxidized CNF followed by the oxidative polymerization of polypyrrole which improved the wettability, mechanical properties, thermal protection, and more importantly, the electrical conductivity by a factor of 105 times [44]. Jiang et al. prepared a PEM from bacterial cellulose through the incorporation of phosphoric acid and phytic acid for improved power density, thermal stability, mechanical strength, and flexibility [45].

More recently, various researchers prepared oxidized nanocellulose-based membranes with various methods and demonstrated the potential for its application in PEMFC. Bayer et al. reported pure nanocellulose membranes in which the proton conductivity increases up to 120 °C and with superior hydrogen barrier properties [36]. The maximum conductivity was 0.05 mS cm^−1^ at 100 °C for the CNF paper membrane and 4.6 mS cm^−1^ at 120 °C for the CNC paper membrane (both at 100% RH), and their power densities at 80 °C were 17 mW cm^−2^ and 0.8 mW cm^−2^, respectively [36]. Jankowska et al. found that all cellulose films showed similar thermal properties from room temperature to about 200 °C. However, the TEMPO-oxidized CNF film showed the highest proton conductivity of the samples studied, including non-oxidized CNF [46]. Guccini et al. evaluated the performance of thin carboxylated CNF-based membranes and obtained an optimized proton conductivity exceeding 1 mS cm^−1^ at 30 °C between 65 and 95% relative humidity (RH), only one order of magnitude lower than Nafion 212, while also exhibiting a lower hydrogen crossover despite being approximately 30% thinner [47]. We also recently achieved 14.2 mS cm^−1^ and a power density of 19.1 mW cm^−2^ at high temperature (80 °C) using nitro-oxidized CNFs with carboxylic acid functional groups [48].

While much progress has been made toward enhancing the proton conductivity of nanocellulose-based PEMs, other problems remain relatively unaddressed. Although its hydrophilic nature provides nanocellulose with excellent gas barrier properties, increasing ionic conductivity has been found to cause excessive water uptake, leading to decreased dimensional stability (i.e., high swelling) [49,50]. While several methods of cross-linking to improve the mechanical properties have been developed, polycarboxylic acids, such as citric acid, have been identified as an effective strategy because of their environmental friendliness [49,51,52,53]. In this study, we aim to enhance wood pulp-derived CNF membrane fuel cells via cross-linkage with citric acid as a “green” method to improve both PEMFC performance and durability.

## 2. Results and Discussion

### 2.1. Citric Acid Cross-Link Characterizations

**Figure 1:**
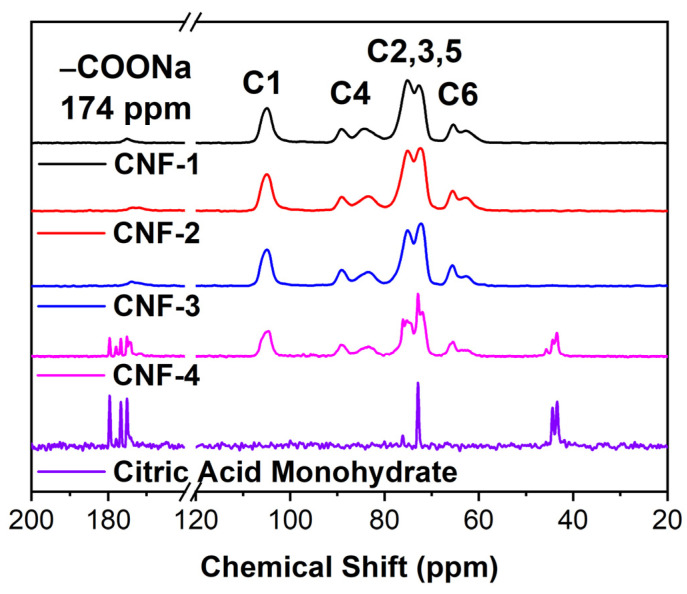
^13^C CPMAS NMR spectra of all CNF membranes and the citric acid monohydrate.

In our study, we characterized CNF membranes with no, low, optimal, and excess citric acid levels, which are denoted as CNF-1 through CNF-4, respectively (see Section 3.3). Solid state ^13^C CPMAS NMR spectroscopy was performed to verify that citric acid chemically cross-linked CNF membranes via the formation of ester bonds between the carboxyl groups from citric acid and hydroxyl groups from CNF fibers [53,54]. In Figure 1, the 60–70 ppm peak was assigned to the C6 carboxylate carbon, the region from 70–80 ppm accounted for C2, C3, and C5 carbons, and the region from 80–93 ppm was assigned to the C4 carbon. As shown in Figure S1, the peak intensity is similar for CNF-1, 2, 3, but with the addition of citric acid, the peak slightly shifted and broadened, which demonstrated citric acid cross-linking of cellulose [55,56]. However, in the spectrum for CNF-4, there were peaks corresponding to citric acid monohydrate peaks, suggesting there is an excess of citric acid in these samples that were not chemically cross-linking nanocellulose. This may arise from the formation of the half-ester intermediate without completing the cross-link if the reaction is diffusion limited. Similarly, at 42–45 ppm and 73 ppm, peaks corresponding to excess citric acid appeared in the spectrum for CNF-4.

**Figure 2:**
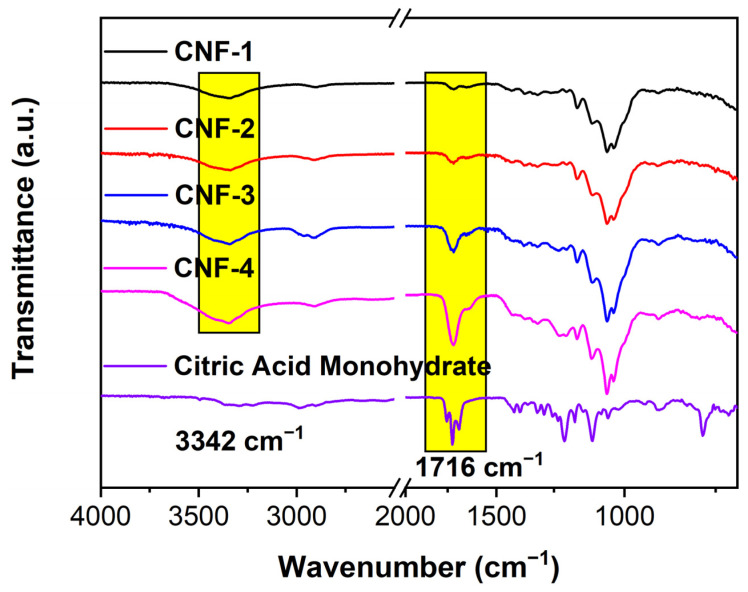
FTIR spectra for CNF membranes with citric acid monohydrate as reference.

Figure 2 shows the FT-IR spectra of the cross-linked CNF membranes plotted with other reference samples. For raw wood and wood pulp, the peak at approximately 1058 cm^−1^ is attributable to the C-O stretching vibration mainly from the cellulose C-O bonds, and this peak is also similarly present in the CNF samples [57,58]. For the uncross-linked CNF and the cross-linked CNF membranes, the peaks at approximately 3342 cm^−1^, 2902 cm^−1^, and 1317 cm^−1^ correspond to the O-H stretching, C-H stretching, and C-H bending, respectively [18,53,54,56,58,59].

Moreover, the intensity of the C=O stretching peak at approximately 1716 cm^−1^ increases from the uncross-linked CNF membranes to the cross-linked CNF membranes with an increase in citric acid addition. The C=O stretching peak of the uncross-linked CNF membrane results from the carboxylic acid (-COOH) groups from TEMPO oxidation of hydroxyl on the C6 position of anhydroglucose units [60]. Given that the citric acid monohydrate also demonstrates a strong peak of C=O stretching in -COOH groups at approximately 1724 cm^−1^, the increasing intensity of C=O vibration likely corresponds to additional -COOH and ester groups formed in citric acid cross-links in the cross-linked CNF membranes [53,54,56,60]. Since the other peaks in crystalline citric acid disappear in the cross-linked CNF spectra, it can be deduced that only molecularly dispersed citric acid exists in the membrane.

**Figure 3:**
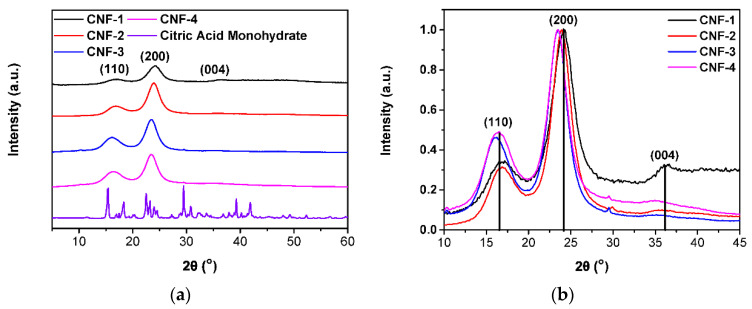
(**a**) Smoothed X-ray diffractometry patterns of CNF membranes and citric acid monohydrate samples. (**b**) Peaks corresponding to cellulose characteristic lattice planes (110), (200), and (004) are indicated in the overlaid diffraction patterns.

From smoothed X-ray diffractometry patterns in Figure 3a, three peaks at around 2θ angles of 16.1°, 23.8°, 35.5° corresponding to the (110), (200), and (004) lattice planes of cellulose, respectively, appear in all the normalized XRD patterns of cross-linked CNF in Figure 3, consistent with the literature [36,57,58]. Slight shifts in the peak location may arise from a slight misalignment of the sample on the stage during data collection. In Figure 3b, the citric acid monohydrate peaks, which align with that in the literature [61], disappear in the cross-linked CNF patterns, demonstrating that citric acid is molecularly dispersed in the CNF membranes. As shown in Table S1, using the “Segal method” for crystallinity estimation [36,62], there is no significant change or trend in crystallinity as citric acid was incorporated since it is a small organic cross-link that does not alter the pre-existing crystalline or amorphous regions of the CNFs, which has been attributed to the cross-link taking place in the amorphous region of CNF [63]. A higher degree of crystallinity may allow superior fuel cell performance as more tightly packed CNFs further reduce hydrogen permeability and crossover through the membrane [36].

**Figure 4:**
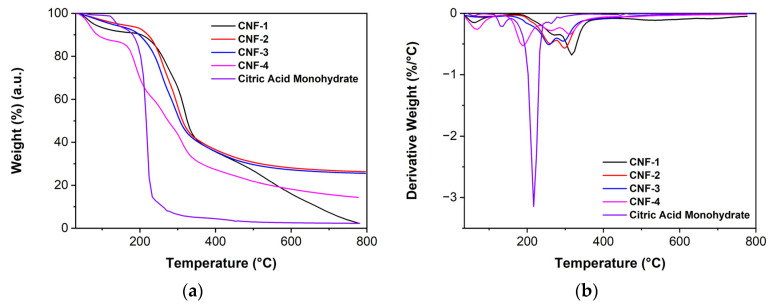
(**a**) TGA and (**b**) DTG graphs of CNF and raw materials.

The thermal stability of the control and cross-linked CNF membranes was assessed by TGA. As shown in Figure 4a, the residual weight of the uncross-linked CNF is relatively insignificant. In contrast, cross-linked CNF membranes exhibited 20–30% residue weight, which results from enhanced carbonization arising from citric acid cross-links that increase the carbon content of the membranes [54]. While we cannot entirely discount the possibility that the citric acid crosslink induces changes in pathways and mechanisms of thermal degradation of CNF, it is less likely to be in action as no significant trend in the CI has been observed with increasing citric acid crosslink as shown in Table S1 in contrast to the previous literature where the decrease in hydrogen bonding reduces CNF reduces crystallinity, thus promoting CNF thermal degradation at lower temperatures [64].

The DTG curves of the samples are shown in Figure 4b. For the cross-linked CNF membranes, the initial mass loss of around 100 °C results from the dehydration of the membrane. In CNF-4, the peak at approximately 190 °C, which corresponds roughly to the mass loss of water from citric acid monohydrate, may be attributed to the citric acid that is in excess and is not chemically bonded to the CNF membranes. The peaks at approximately 250 °C and 300 °C correspond to the degradation of the anhydroglucoronic and anhydroglucose units in CNF, respectively [58].

In addition, for CNF-4, the degradation peak at approximately 190 °C can be observed but is absent in the CNF samples with lower citric acid content. This peak shares a similar onset temperature (approximately 170 °C) with the second degradation step of citric acid monohydrate. Thus, this degradation peak may be attributed to the degradation of the excess citric acid present in the CNF samples with a higher citric acid content [54,65]. Thus, the citric acid cross-link has reached saturation in CNF-4, and the excess citric acid exists as residual molecules adsorbed to the solvent-casted membrane. Importantly, all degradation onset temperatures are well above typical proton exchange membrane fuel cell operating conditions of 80 °C.

### 2.2. Morphology Characterizations

**Figure 5:**
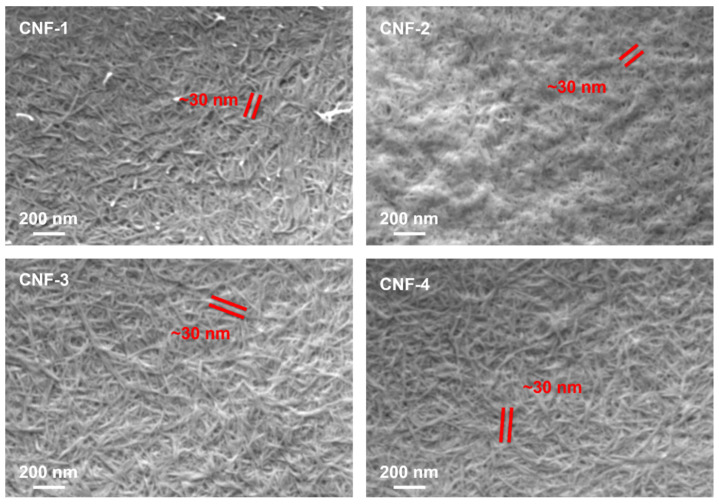
CNF membrane SEM images of the surface-exposed side during solvent casting. Red lines indicate typical thickness (~30 nm) of CNF fibers in corresponding membranes.

Figure 5 displays surface SEM images of the surface-exposed side from the solvent-casting process. There is no micron-scale porosity, and the fiber dimensions do not appear to change with the addition of citric acid. The membranes are rough from the evaporation process in solvent-casting. EDX imaging is in the Supplementary Information (Figure S2).

**Figure 6:**
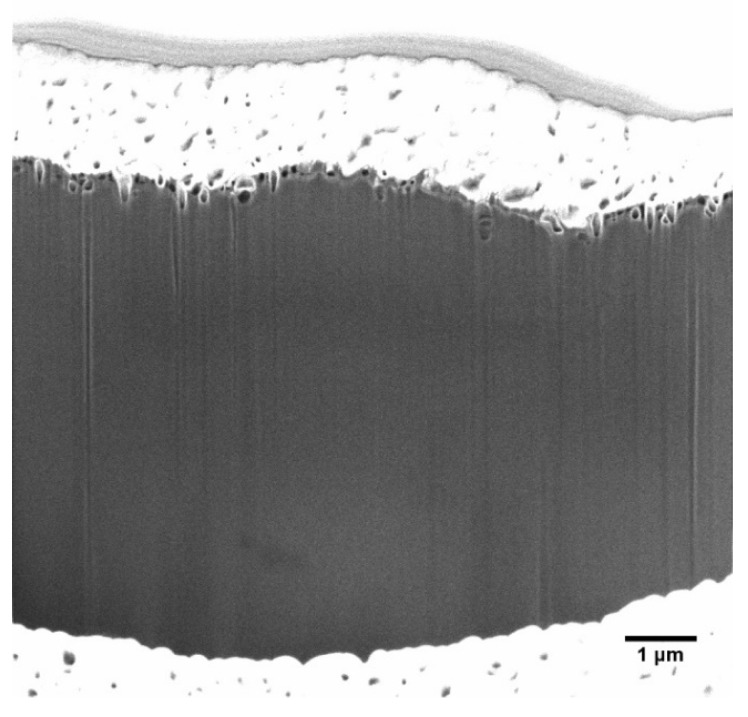
10 × 10 μm^2^ FIB/SEM image of a diagonal corner cross-section of CNF-4.

In Figure 6, a 10 × 10 μm^2^ diagonal cross-section through a corner of the CNF-4 film is displayed. Notably, the interior of the CNF film is uniformly dense, with no porosity on the micron scale. The dark streaking results from beam damage, which is expected in organic materials, while the white layers at the top and bottom are the palladium sputter coat, and the grey above the sputter coat is from charging.

**Figure 7:**
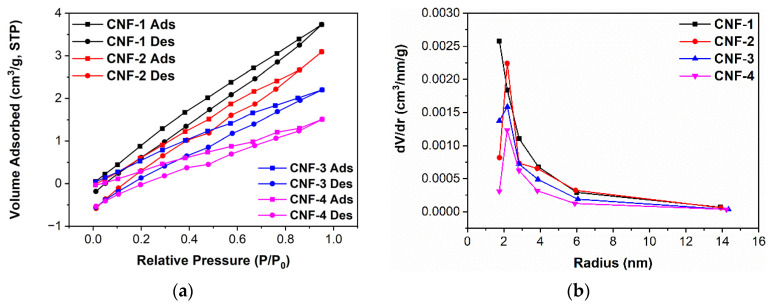
(**a**) BET adsorption (Ads) and desorption (Des) isotherms, (**b**) pore size distribution based on BJH analysis.

The porosity of the membranes was assessed by nitrogen adsorption and desorption. Figure 7a suggests a low nanoscale porosity in the internal morphology of CNFs, as nitrogen adsorption is linear with relative pressure, which occurs with adsorption to only the surface of the sample. The individual BET isotherms with linear fit are shown in Figure S3. As shown in Table 1, the BET surface area and total pore volume of CNF membranes demonstrate a decreasing trend with the increase in citric acid cross-linking, which is in good agreement with previous literature [66,67,68]. The pore size distributions based on BJH desorption analysis are displayed in Figure 7b, and it can be observed that the citric acid cross-link does not alter the average pore size of the CNF samples significantly. Overall, the already low porosity decreases further with the addition of citric acid, which is desirable in fuel cell membranes as it reduces hydrogen gas crossover and increases fuel cell performance [15,36,47].

### 2.3. Surface Characterizations

**Figure 8:**
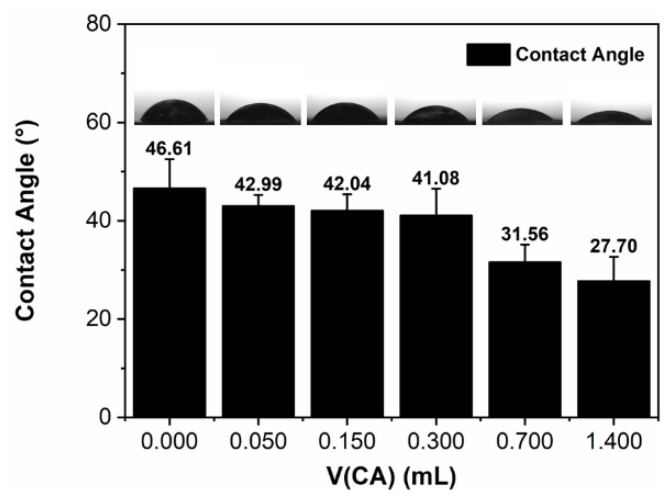
Water contact angle data for all CNF membranes with *n* = 3 and mean written above bars.

Additional membranes of intermediate citric acid levels (0.150 mmol, 0.700 mmol) were synthesized for investigation of fuel cell performance trends. The hydrophobicity of a membrane can be determined through the water contact angle: the larger the angle, the more hydrophobic the membrane is, and vice versa. Figure 8 shows triplicate contact angle measurements for all CNF membranes. The measurements show a trend of decreasing hydrophobicity of the films as citric acid addition increases. In particular, the initial addition of 0.050 mL of 1 M citric acid resulted in a 9.8% decrease from 46.61° to 42.99°. This decrease in contact angle results from the increased polarity of cross-linked CNF, which evidences the incorporation of citric acid and its highly polar carboxyl groups and ester groups. While three carboxyl groups are present per citric acid molecule, it is not necessary for all three to participate in crosslinking, thus introducing additional free carboxyl groups into the CNF membranes. More carboxyl groups facilitate an increase in fuel cell performance, as negatively charged tunnels are the primary way protons permeate through the membrane [35,43].

**Figure 9:**
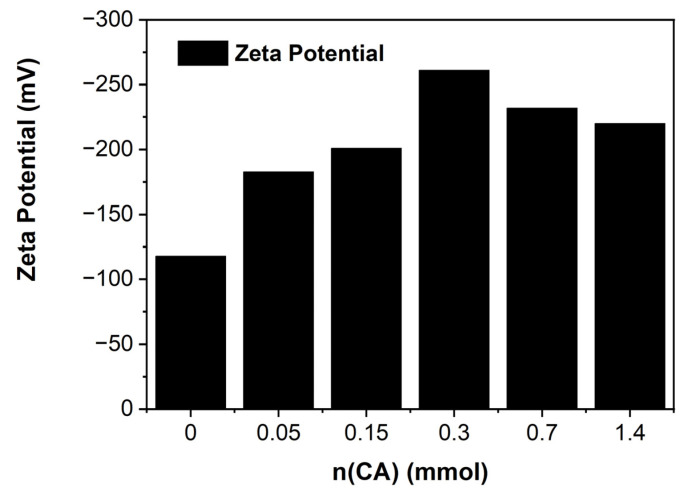
Zeta potential at pH = 7.0 for all CNF membranes.

The surface charge of the CNF samples was determined through zeta potential measurements at a neutral pH, shown in Figure 9. Initially, with the addition of citric acid, the surface charge increases from the additional negatively charged carboxyl group until 0.300 mmol (CNF-3). Initially, the three carboxyl groups for citric acid are not necessarily fully esterified, leading to an increase in the negative zeta potential. After a saturation point, the surface charge decreases with further addition beyond 0.300 mmol. This may be explained by the process where part of the citric acid starts to be fully esterified, which does not contribute negatively charged carboxylate groups, while the excess unreacted citric acid was evidenced by the excess citric acid peaks in TGA as shown in Figure 4.

### 2.4. Fuel Cell Performance

**Figure 10:**
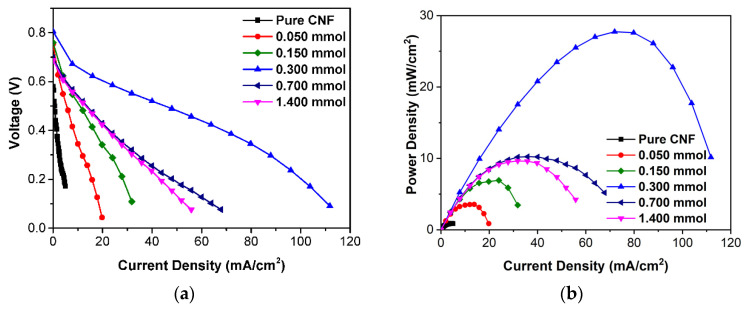
(**a**) Polarization curves and (**b**) power density curves of all membranes.

**Figure 11:**
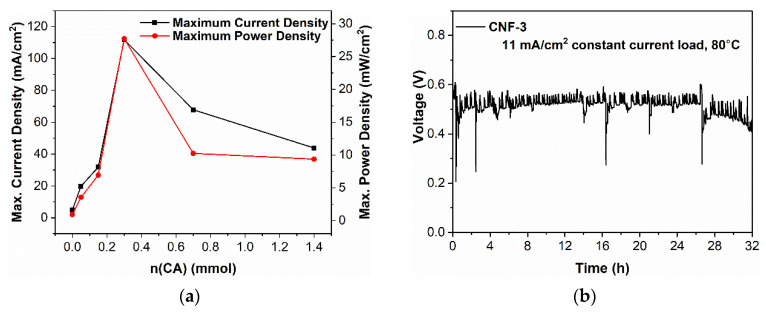
(**a**) Trend of maximum current density and power density with citric acid addition. (**b**) 24 h constant current density load durability test.

Additional cross-linked CNF membranes with 0.150 mmol and 0.700 mmol citric acid were synthesized for the optimization of fuel cell performance. The cross-linked CNF membrane MEAs were evaluated on the fuel cell test station and the resulting polarization and power density curves are shown in Figure 10a,b, respectively. The control CNF-1 membrane had an open circuit voltage (OCV) of 0.58 V. While this value is lower than previously reported (32 μm thickness, 0.97 V), the CNF membrane as prepared here is significantly thicker (~75 μm thickness), thus increasing resistance and decreasing the OCV [36,47]. On the other hand, the cross-linked CNF membranes demonstrate a significantly enhanced OCV of 0.75 V compared to the uncross-linked CNF-1, which results from the citric acid cross-linkage creating negatively charged conduction tunnels for protons that lower resistance and increase the OCV. At 80 °C and 100% RH, the maximum current and power densities obtained for each membrane are shown in black in Figure 11a, with the highest maximum current density of 111.8 mA cm^−2^ and power density of 27.7 mW cm^−2^ being obtained with CNF-3 (0.300 mmol citric acid). Compared to the uncross-linked CNF-1, which had a maximum current density of 5.0 mA cm^−2^ and a maximum power density of 0.91 mW cm^−2^, CNF-3 achieved an approximately 2200% increase in maximum current density and 3000% increase in maximum power density.

In Figure 11a, with the increase in citric acid, there was an initial improvement in performance parameters up until 0.300 mmol of citric acid. Beyond 0.300 mmol, the performance declined with further citric acid addition. This reflects the trend in zeta potential, whereby negative surface charge increases with citric acid addition until 0.300 mmol before decreasing with further addition. Thus, the decrease in performance with excess citric acid addition can be explained by the loss of negatively charged groups. Additionally, previous literature found a similar effect of increasing the amount of acid dopant in bacterial cellulose membranes, as the excess dopant phase separates and reduces the degree of freedom of ion transport and proton mobility [45,69]. Figure 11b shows the long-term durability test on CNF-3, the best-performing membrane with 11 mA cm^−2^ constant current density load at 80 °C, which remains relatively stable for at least approximately 30 h, demonstrating excellent stability compared with previous literature [36,47].

**Figure 12:**
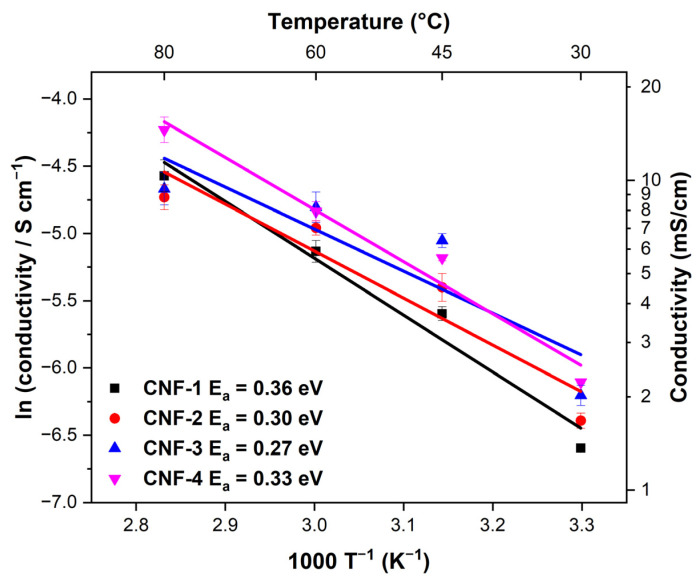
Arrhenius plots and activation energies of CNFs at 100% RH.

Since additional carboxylic acid groups contribute to the proton transport mechanism, we investigated the effect of increasing the quantity of citric acid in the cross-linked membranes on proton conductivity. Figure 12 shows the Arrhenius plots of the CNF membranes. As expected, for all membranes, increasing the temperature from 30 °C to 80 °C continuously increased the conductivity by nearly an order of magnitude. The activation energies were determined from the linear Arrhenius law fit. A similar trend to the fuel cell performance can be found, with the lowest activation energy (0.27 eV in CNF-3) corresponding to the highest maximum power density and current density. For CNF-3, a maximum of 9.4 mS cm^−1^ is obtained at 80 °C. The activation energies were higher than that of the Nafion (E_A_ = 0.16 ± 0.02 eV), but are still in good agreement with the Grotthuss-like, water-mediated mechanism previously proposed for carboxyl cellulose films (0.1–0.4 eV), which features a lower activation energy than the vehicle transport mechanism dominant at lower humidity (0.5–0.9 eV) [36,70,71,72]. Another possible transport mechanism is the proton-hopping process along the oxygen-containing functional groups of the cellulose, similar to the proton-hopping process between the -SO_3_^2-^ groups on the surface in Nafion proposed in previous literature [36,73]. It is likely that the citric acid not only cross-links the membrane, but also contributes extra negatively charged oxygen groups to enhance the proton hopping and thus decrease the activation energy. As shown in Table 2, our Citric acid cross-linked CNF membranes demonstrate significantly improved power densities over literature precents utilizing a variety functionalized cellulose nanofibers [36,42,45,48]. Higher performances with “green” CNF membranes might be achieved by exploring other cross-linking agents with more extensive carboxyl groups that would facilitate enhanced proton conduction while also improving the mechanical properties of the membrane.

**Figure 13:**
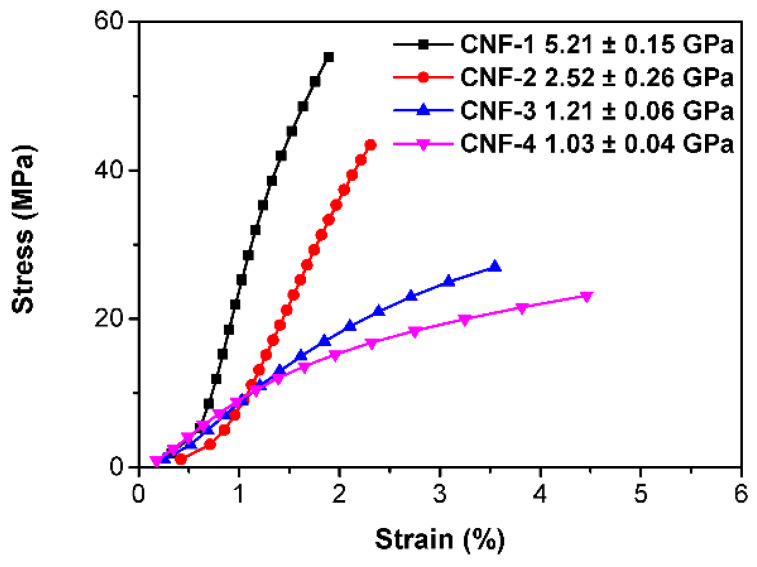
Dry membrane tensile strength DMA.

Dry CNF membranes were evaluated by DMA tensile strength testing, and the stress–strain curves are shown in Figure 13. The addition of citric acid lowered the breaking strength and elastic modulus while increasing elongation. The cross-link plasticized the CNF, which is consistent with previous literature. It has been suggested that citric acid cross-linking would reduce rigidness, increase flexibility, facilitate hydrogen bonding networks, increase free volume, improve thermal stability, and increase the proton conductivity of polymers, making them suitable for applications in PEMFC [15,45,53,68,74,75,76,77]. This corroborates the improved power density as well as the improved durability over time under constant current density load in the fuel cell test station compared with uncross-linked membranes in previous literature [36], and demonstrates the potential for further engineering.

## 3. Materials and Methods

### 3.1. Materials and Chemicals

In this study, CNF was derived from delignified wood pulp. All reagents used in the TEMPO oxidation process were analytically pure and used as purchased. Carbon paper electrodes with 0.1 mg/cm^2^ Pt loading were purchased from FuelCellsEtc (College Station, TX, USA). H_2_, N_2_, and Air were purchased from Airgas (Radnor, PA, USA).

### 3.2. Preparation of Carboxycellulose Nanofibers

Cellulose nanofibers were prepared from wood pulp via the TEMPO oxidation process according to previous literature [78,79]. In this process, 10.0 g delignified wood pulp was well dispersed in about 500 mL of DI water. NaBr (1.0 g) and TEMPO reagent (0.20 g) were subsequently added into the dispersion stirrer for 15–20 min to make it homogeneous. The pH value of the reaction mixture was maintained at 10.0 during the reaction process by the slow addition of 1 M NaOH solution. The oxidation process was initiated by adding 112.0 g NaOCl under continuous stirring for 20 h. There were frequent pH changes observed at the initial stages of the experiment, which is due to a fast reaction, but fluctuations became less noticeable after 3–4 h. The reaction was quenched by adding 100 mL ethanol solution and stirring vigorously for 20 min. Cellulose fibers were separated by centrifugation at 7000 rpm and washed 3 times with DI water. Finally, the CNF was dialyzed until the conductivity of the suspension was 5 μS. The final concentration of the bulk CNF suspension was measured to be 0.35 wt %.

### 3.3. Preparation of Citric Acid Cross-Linked Carboxycellulose Nanofiber Membranes

The CNF suspension was diluted to 0.20 wt %. The citric acid cross-linked carboxycellulose nanofibers were prepared by adding X mL of 1.0 M citric acid solution into 70.0 mL of CNF suspension (X = 0.000, 0.050, 0.300, 1.400) and left to soak overnight. The resulting membranes with no, low, optimal, and high citric acid levels are denoted as CNF-1 through CNF-4, respectively. Cross-linked CNF membranes were prepared by the solvent evaporation method. The cross-linked CNF suspension was transferred to a glass Petri dish and dried at 70 °C into a thin membrane. The membranes were further dried under a hot press at 110 °C (approximately 230 °F) for 600 s. A membrane of CNF without citric acid is synthesized in a similar method without the addition of citric acid solution.

### 3.4. Fourier-Transform Infrared-Red Spectrometry (FTIR)

A Perkin Elmer Frontier FT-IR spectrometer with an attenuated total reflectance (ATR) accessory was used to record FT-IR transmission spectra from 700 to 4000 cm^−1^. A total of 32 scans were averaged, and the resolution was 2 cm^−1^.

### 3.5. Thermogravimetric Analysis (TGA)

The thermal stabilities of the wood pulp and derived cross-linked CNF membranes were evaluated using a Mettler Toledo TGA/SDTA851e instrument. Both TGA and differential thermogravimetry (DTG) curves were collected under nitrogen flow from 35–800 °C at a heating rate of 10 °C/min.

### 3.6. ^13^C CPMAS NMR

Solid state ^13^C CPMAS NMR spectroscopy of cross-linked CNF membranes was conducted on a Bruker Ultrashield 500WB plus (500 MHz) NMR instrument. The probe was a 2.5 mm triple resonance magic angle spinning (MAS) probe, which spins samples with frequencies up to 35 kHz. The resonance frequency for ^13^C was 10,000 Hz, and the cross-linked CNF samples were spun at the magic angle at 10 kHz.

### 3.7. X-ray Diffractometry (XRD)

Crystallinity was also determined using a Rigaku Miniflex diffractometer with Cu Kα radiation (1.5406 Å, 0.08°/step, data collection from 5 to 60°). The crystallinity index for each sample was calculated using the following equation [36,62], where I_total_ is the intensity of both crystalline and amorphous parts (peak intensity ~23.4°) and I_amorphous_ is the intensity of the amorphous part (minimum intensity ~19.8°) of the sample.

(1) $$\mathrm{CI}=\frac{I_{\mathrm{total}}-I_{\mathrm{amorphous}}}{{\;I}_{\mathrm{total}}}\times 100\%$$

### 3.8. Focused Ion Beam Scanning Electron Microscopy (FIB/SEM)

The internal morphology of the cross-linked CNF membrane was investigated with a ZEISS Crossbeam 340 FIB/SEM instrument. To mitigate charge build up, the samples were sputter-coated with Pd. The FIB acceleration voltage was set to 30 kV and the emission current was 2 μA. A 10 × 10 μm area of a diagonal cross-section through a corner was imaged.

### 3.9. BET Surface Area

The specific surface areas of freeze-dried cross-linked CNF membranes were determined by N_2_ adsorption at 77 K using a Quantachrome NOVAtouch LX2 Brunauer-Emmett-Teller (BET) analyzer. The analyzer performed both degassing and BET analysis functions. The ~0.10 g samples were degassed under dry N_2_ flow at 100 °C for 12 h prior to analysis. The samples were then loaded into a 9 mm bulb end cell and a BET adsorption–desorption isotherm was recorded in the presence of a reference cell.

### 3.10. Transmission Electron Microscopy (TEM)

Cross-linked CNF suspensions were studied with an FEI Tecnai G2 Spirit BioTWIN instrument equipped with a digital camera. The accelerating voltage was 120 kV. For each sample, 10 µL of 0.01 wt % cross-linked CNF suspension was deposited on freshly glow discharged carbon coated 300 mesh Cu grids from Ted Pella Inc. and stained with 2 wt % aqueous uranyl acetate solution.

### 3.11. Surface Scanning Electron Microscopy with Energy Dispersive X-ray Analysis (SEM/EDX)

Membrane morphology was investigated with a Zeiss LEO 1550 SFEG-SEM instrument equipped with an In-Lens Secondary Electron Detector, a standard E-T detector, and a Rutherford Backscatter Electron Detector. The instrument was also equipped with an EDS (energy dispersive X-ray spectroscopy) system using an EDAX detector, which provided elemental compositions and X-ray maps of the various phases of the materials examined.

### 3.12. Contact Angle

Static water contact angles of cross-linked CNF films were measured by a KSV CAM 200 optical tensiometer. Room temperature 5 μL water droplets were deposited on the membranes by micropipette, and the contact angle of each membrane was measured 20 s after drop deposition to ensure the water droplet reached equilibrium. Each membrane was evaluated in triplicate to account for surface inhomogeneity.

### 3.13. Fuel Cell Testing

The membrane single-cell performance was tested in a fuel cell test station from Fuel Cell Technologies, Inc. Both gas diffusion layer electrodes were commercial carbon cloth loaded with 0.1 mg/cm^2^ Pt. The membrane electrode assembly (MEA) was assembled by uniformly compressing the membrane between the electrodes such that pressure was evenly distributed across the MEA. The MEA active area was 5 cm^2^. The tests were conducted with flow rates of 100 sccm 99.99% H_2_ at the anode and 100 sccm 99.99% pure O_2_ at the cathode. At both electrodes, the gases were preheated to 85 °C to prevent condensation and humidified to 100% relative humidity (RH). The fuel cell operating temperature was 80 °C. The performance stability of CNF-4 was determined by running the test station at 80 °C under constant current density (11 mA/cm^2^) for 40 h.

### 3.14. Dynamic Mechanical Analysis (DMA) of Cross-Linked CNF Membrane

The mechanical strengths of the cross-linked CNF membranes were assessed using a TA DMA Q800 instrument. Stress–strain measurements were taken on a ~10 × 1 × 0.05 mm (L × W × T) sample. The tension clamp preload was 0.03 N and then tension was increased at a rate of 3 N/min until the sample fractured. Dry samples were measured.

### 3.15. Zeta Potential

The zeta potentials of cross-linked CNF samples were measured by a Zetaprobe Analyzer™ from Colloid Dynamics, Ponte Vedra Beach, FL, USA. The instrument used a built-in titration setup equipped with a pH electrode and an ESA sensor. Before analysis, the pH electrode was calibrated using pH buffers standards (pH = 4.01, 7.01, and 10.01), followed by a standard titration solution. The ESA sensor was calibrated with the standard zeta probe polar solution (KSiW solution). After calibration, 100 mL of 0.20 wt % cross-linked CNF suspension was transferred to the sample holder, where the ESA sensor was placed in with magnetic stirring to analyze the zeta potential at neutral pH.

## 4. Conclusions

In order to improve upon the performance of nanocellulose for the application as a green and low-cost proton-exchange membranes in hydrogen fuel cells, citric acid is introduced as a cross-linking agent to improve both the proton conductivity and the mechanical strength simultaneously. Citric acid cross-linked nanocellulose membrane were synthesized by solvent-casting and we determined the optimal range of citric acid addition for fuel cell performance. Chemical cross-linkage with citric acid was demonstrated with ^13^C NMR, FT-IR spectroscopy, and XRD. The cross-linked CNF membranes is shown to have high density, low porosity, and unaffected crystallinity with citric acid addition, giving the membranes excellent hydrogen barrier properties.

Polarization and power density curves were recorded for cross-linked CNF membrane MEAs operated in oxygen with 0.1 mg cm^−2^ Pt loading at the anode and cathode. CNF-3 exhibited a maximum current density of 111.8 mA cm^−2^ (2200% increase compared to the control) and a maximum power density of 27.7 mW/cm^−2^ (3000% increase compared to the control), and the optimal quantity of citric acid addition lies between 0.150 and 0.700 mmol citric acid with 70 mL CNF suspension. From the proton conductivity measurements, there is a corresponding minimum activation energy of 0.27 eV achieved concurrently with the performance optimum, and a maximum of 9.4 mS cm^−1^ is obtained at 80 °C. The long-term durability lasted more than approximately 30 h under an 11 mA cm^−2^ load, while the citric acid acts as a plasticizer to reduce elastic modulus and enhance the flexibility and durability of the membrane. To the best of our knowledge, this work provides the first example where the simple addition of citric acid as a cross-linking agent is proposed as a low-cost method to improve both fuel cell performance and durability, and to inspire future research and applications of nanocellulose proton-exchange membranes in hydrogen fuel cells.

## Figures and Tables

**Table 1:** Surface area and total pore volume of CNF membranes based on BET absorption/desorption isotherm analysis.

	Surface Area (m^2^/g)	Pore Volume (cc/g)
CNF-1	1.63720	0.0057850
CNF-2	1.26727	0.0047933
CNF-3	0.94770	0.0034088
CNF-4	0.64708	0.0023407

**Table 2:** Comparison of maximum power density of CNF-3 with cellulose-based PEM in previous literature. [36,42,45,48].

Reference	PEM Material	Maximum Power Density (mW cm^−2^)
Jiang et al., 2012 [45]	Phytic acid-incorporated Bacterial Cellulose	23.0
	Phosphoric acid-incorporated Bacterial Cellulose	17.9
Bayer et al., 2016 [36]	Cellulose Nanocrystal	17.2
	Cellulose Nanofiber	0.8
Wang et al., 2019 [42]	Nafion-impregnated Cellulose Filter Paper	23
	RDP-impregnated Cellulose Filter Paper	10
Sharma et al., 2022 [48]	Nitro-oxidized CNF w/Carboxylic Acid Functionalities	19.1
	Nitro-oxidized CNF w/Carboxylate Functionalities	5.8
This Study	Citric acid cross-linked CNF	27.7

## Supplementary Materials

The supporting information can be downloaded at: https://www.mdpi.com/article/10.3390/ijms232315245/s1.
