# Supplementary material for: Sustainable Plant-Based Biopolymer Membranes for PEM Fuel Cells
Source: Int J Mol Sci. 2022 Dec 3;23(23):15245. doi: 10.3390/ijms232315245 (PMC9741098; doi:10.3390/ijms232315245)
Supplement: Supplementary file 1 [file ijms-23-15245-s001.zip › ijms-2053244-supplementary.pdf]

International Journal of
Molecular Sciences

MDPI

Article

Sustainable Plant-Based Biopolymer Membranes for PEM Fuel Cells

Supplementary Materials

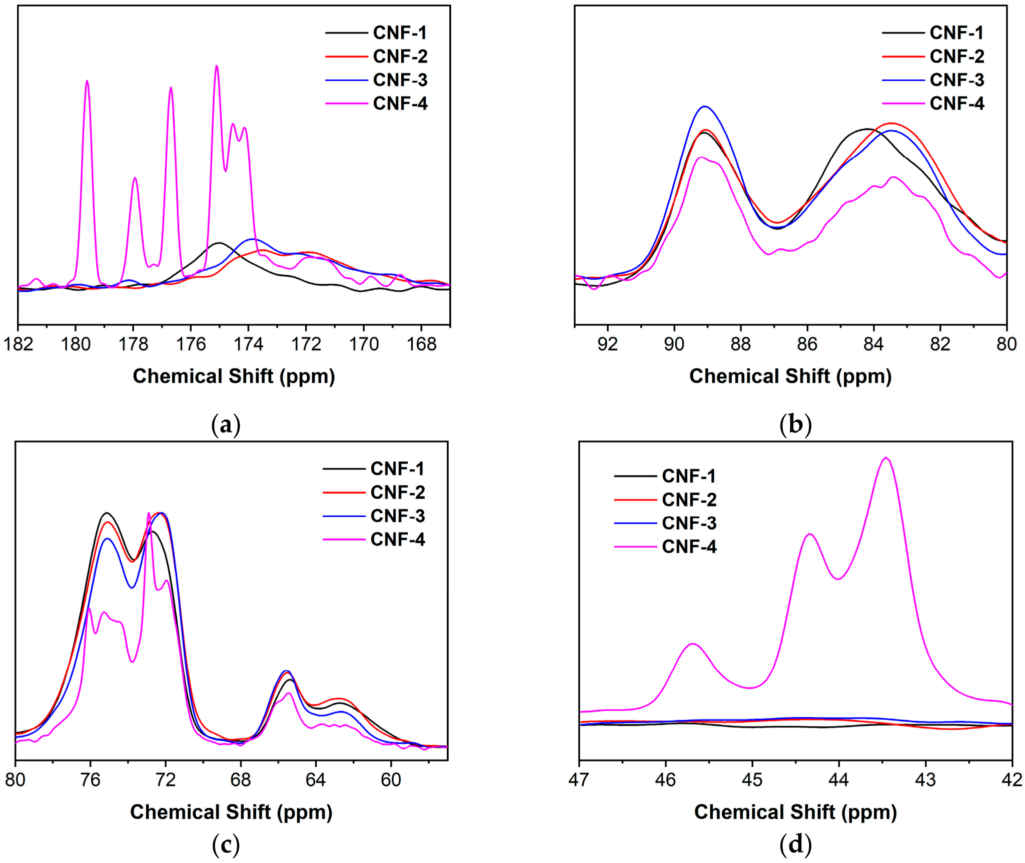

Figure S1. Zoom in of ^{13}C CPMAS NMR spectra of CNF membranes (a) 182-167 ppm, (b) 93-80ppm, (c) 80-57 ppm, (d) 47-42 ppm.

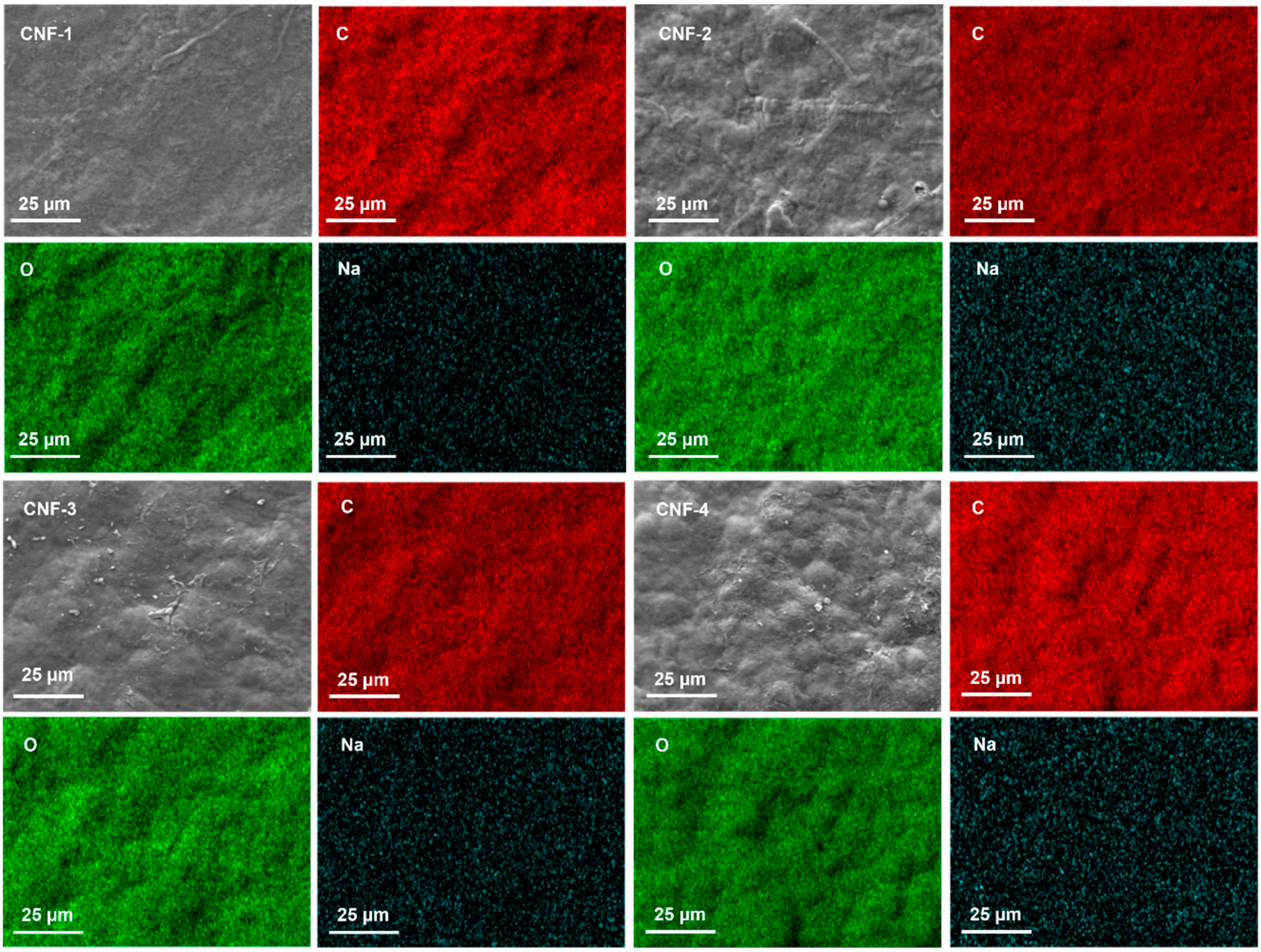

Figure S2. CNF membrane SEM and EDX images of the surface-exposed side during solvent casting.

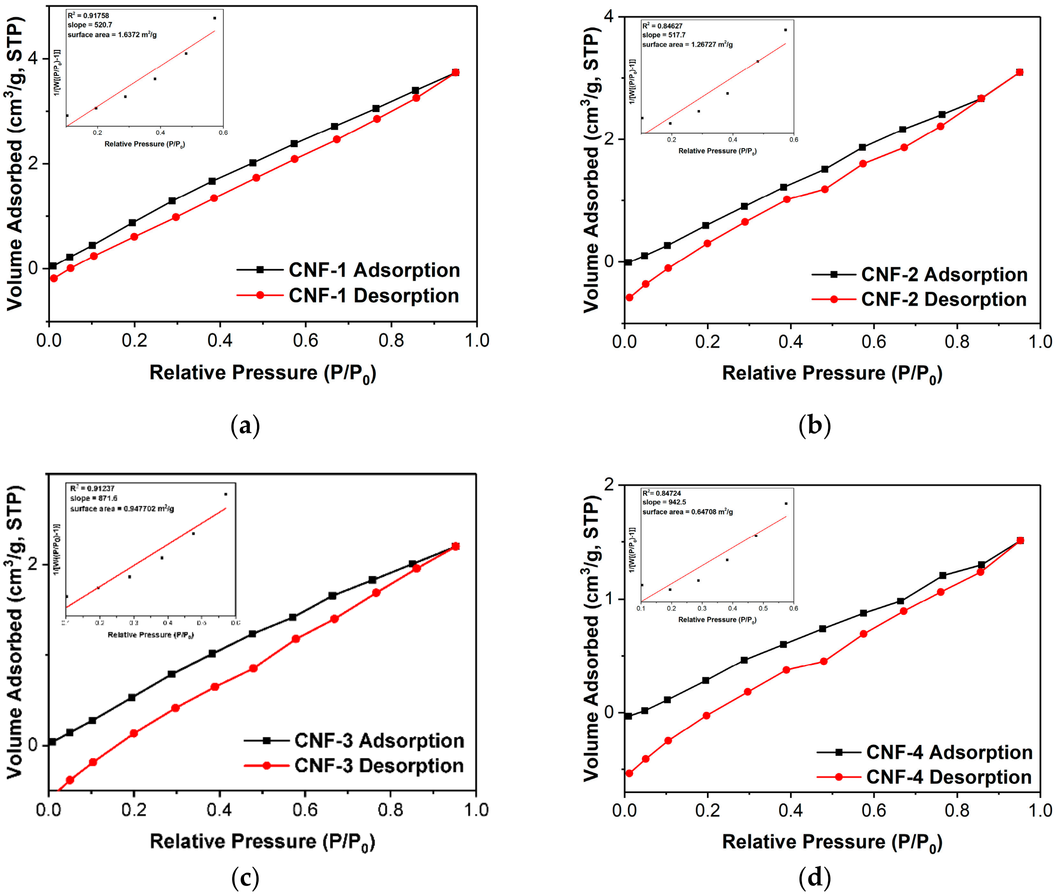

Figure S3. Individual BET isotherms with linear fit (inset) of CNF membranes, (a) CNF-1, (b) CNF-2, (c) CNF-3, (d) CNF-4.

Table S1. Crystallinity Index (CI) of CNF membranes estimated by "Segal Method".

	Crystallinity Index
CNF-1	76%
CNF-2	74%
CNF-3	80%
CNF-4	75%
